# Phylogeographic history of South American populations of the silky anteater *Cyclopes didactylus* (Pilosa: Cyclopedidae)

**DOI:** 10.1590/1678-4685-GMB-2016-0040

**Published:** 2017-02-13

**Authors:** Raphael Teodoro Franciscani Coimbra, Flávia Regina Miranda, Camila Clozato Lara, Marco Antônio Alves Schetino, Fabrício Rodrigues dos Santos

**Affiliations:** 1 Laboratório de Biodiversidade e Evolução Molecular, Departamento de Biologia Geral, Instituto de Ciências Biológicas, Universidade Federal de Minas Gerais, Belo Horizonte, MG, Brazil.

**Keywords:** Xenarthra, mitochondrial DNA, population structure, molecular dating, South America

## Abstract

*Cyclopes didactylus*, commonly called silky anteater, is the smallest and least studied of the anteaters. It is an arboreal species occurring in rainforests, ranging from southern Mexico to Central and South America, with an apparently disjoint distribution between Amazon and Atlantic rainforests in Brazil. Although seven subspecies are recognized, little is known about its geographical variation. Thus, to evaluate the population dynamics and evolutionary history of the South American silky anteater, we analyzed 1542 bp sequences of the mitochondrial control region (CR), *COI* and *Cyt-b* genes of 32 individuals. Haplotype network, AMOVA and molecular dating analyses were performed and identified seven geographic clusters. The split of lineages separating Cyclopedidae *(Cyclopes)* and Myrmecophagidae (*Myrmecophaga* and *Tamandua* genera) was estimated around 41 million years ago (mya), and the intraspecific lineage diversification of *C. didactylus* began in the Miocene around 13.5 mya, likely in southwestern Amazonia. Tectonic and climatic events that took place in South America during the Tertiary and Quaternary seem to have influenced the evolutionary history of the species at different levels. This is the first study to investigate the population dynamics and phylogeography of the silky anteater, which contributes to a better comprehension of the biogeography of South America.

## Introduction

*Cyclopes didactylus*, commonly called silky anteater, is the only living species of the Cyclopedidae family and the smallest of all known anteaters with adults averaging 430 mm long and 235 g of weight (Gardner, 2007). It has nocturnal habits and appears to be completely arboreal (Montgomery, 1985). This species exhibits a “dense, woolly to silky, silvery-gray to golden-brown body pelage, two digits on the manus and four on the pes, a prehensile tail” (Gardner, 2007) and a “hind feet highly modified for grasping small twigs while climbing and feeding” (Wetzel, 1985).

**Figure 1:**
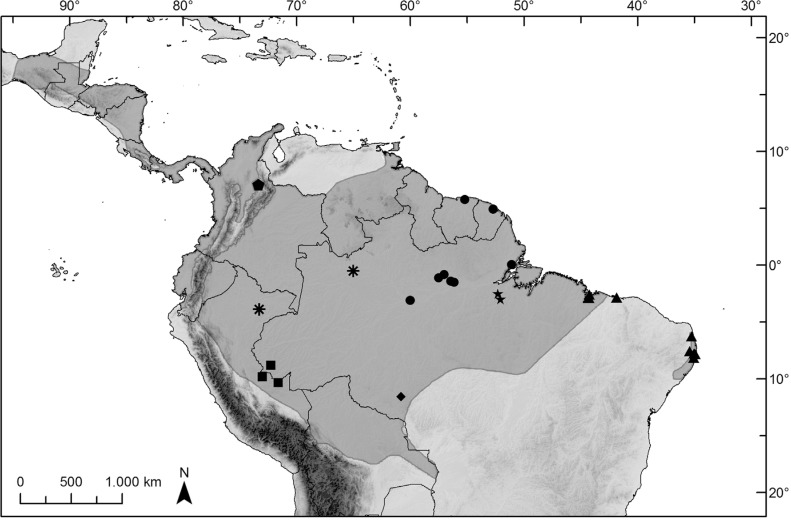
Map of *Cyclopes didactylus* range and sampling localities. Species distribution based on Superina *et al.* (2010). Different symbols mark localities of each geographic clusters. Square = UA; diamond = RO; asterisk = LS; star = PV; pentagon = CWA; circle = MOSF; and triangle = NB.

The silky anteater inhabits tropical rainforests, ranging from southern Mexico to Central and South America, and is apparently disjoint between Amazon and Atlantic rainforests in Brazil (Figure 1; Gardner, 2007). In South America it occurs in the northern Andean valleys of Colombia, in the west of the Andes along the Pacific coast lowlands of Colombia and Ecuador, in the rainforests of Venezuela and Guianas, and southwards into the Amazon basin drainage of the lowlands of Colombia, Ecuador, Peru, Brazil and Bolivia (Gardner, 2007; Miranda and Superina, 2010; Superina *et al.*, 2010). The Atlantic Forest of the northeastern coast of Brazil harbors a small and apparently isolated population, ranging from the states of Rio Grande do Norte to Alagoas (Miranda and Superina, 2010). Considering its wide distribution, little is known of its geographical variation (Aguiar and Fonseca, 2008).

Seven subspecies of the silky anteater are currently recognized, which is based mainly on coat color and the presence of darker dorsal, sternal or ventral stripes (Wetzel, 1982; Gardner, 2007; Hayssen *et al.*, 2012). Among them, *C. d. mexicanus* is the only one that does not occur in South America, while *C. d. dorsalis* is present mainly in Central America but also occurs in northern and northwestern Colombia (Wetzel, 1982; Gardner, 2007; Hayssen *et al.*, 2012). The last subspecies found in the west of the Andes is *C. d. eva*, which occurs in the Pacific coast lowlands of Colombia and Ecuador (Wetzel, 1982; Gardner, 2007; Hayssen *et al.*, 2012). All the other subspecies are located in the east of the Andean Cordillera including *C. d. didactylus*, which is present from Venezuela through Guianas and in northeastern Brazil; *C. d. melini*, occurring in the northern Amazon basin of Brazil and adjoining Venezuela and Colombia; *C. d. ida*, which is found throughout the western Amazon basin; and *C. d. catellus*, occurring in southeastern Peru, northeastern Bolivia and central Amazon basin (Wetzel, 1982; Gardner, 2007; Hayssen *et al.*, 2012).

There is no fossil record for the silky anteater (McDonald *et al.*, 2008), but *Palaeomyrmidon incomptus*, a fossil taxon from the Huayquerian period (9-6.8 mya), is considered its sister group (Hirschfeld, 1976; Gaudin and Branham, 1998). Moreover, Delsuc *et al.* (2004, 2012), based on the analysis of nuclear and mitochondrial data from all living xenarthran genera, estimated the divergence between Cyclopedidae (*Cyclopes*) and Myrmecophagidae (*Myrmecophaga* and *Tamandua* genera) at around 40 and 45.5 mya (middle Eocene), respectively. However, a more recent estimate by Gibb *et al.* (2016) that used complete mitogenomes from all living xenarthran species set it at ca. 37.8 mya.

Home to the origin and diversification of all xenarthran species (Patterson and Pascual, 1972), the South American continent has undergone several geological changes during the Tertiary and Quaternary. Major phases of Andean uplift started in the Paleocene and the most intense peaks of mountain building in the Northern and Central Andes took place in the last 30 million years (Sempere *et al.*, 1990, 1994; Lundberg *et al.*, 1998; Hoorn *et al.*, 2010; Antonelli and Sanmartín, 2011). Episodes of marine incursions from the Pacific (Lundberg *et al.*, 1998; Antonelli *et al.*, 2009), the Caribbean Sea (Hoorn, 1993; Lovejoy *et al.*, 2006) and the Paraná River basin (Lundberg *et al.*, 1998) were recorded from the Paleocene until the Miocene (Lovejoy *et al.*, 2006). Periods of thermal optimum occurred in the early Eocene, late Oligocene and middle Miocene (Zachos *et al.*, 2001) while global cooling trends were documented from middle Eocene to middle Oligocene and since the late Miocene (Zachos *et al.*, 2001). The uplift of the Panama Isthmus triggered the Great American Biotic Interchange (GABI) by ca. 3.5 mya (Hoorn *et al.*, 2010; Antonelli and Sanmartín, 2011). However, recent studies suggest a starting for dispersal pulses of the GABI as early as the Oligocene-Miocene transition (Bacon *et al.*, 2015), and at least a partial closure of the Central American seaway by 13-15 mya (Montes *et al.*, 2015). Finally, the Pleistocene glaciations started ca. 2.6 mya (Hoorn *et al.*, 2010; Antonelli and Sanmartín, 2011), causing many changes in the South American rainforest distribution (Oliveira *et al.*, 1999; Auler *et al.*, 2004; Wang *et al.*, 2004; Ortiz-Jaureguizar and Cladera, 2006). All these tectonic and climatic events during the Tertiary and Quaternary changed the landscapes on the continent and some of them were previously associated with synchronous diversification events in Xenarthra (Delsuc *et al.*, 2004).

To date there is no genetic study regarding population structure and dynamics, and timing of intraspecific lineage diversification for the silky anteater. Here we present the first attempt, using three mitochondrial fragments, to assess the phylogeographic patterns and date the divergences of the South American populations of *Cyclopes didactylus* to compare them with environmental changes occurring in the continent at different times.

## Materials and Methods

### Sample collection and DNA extraction

Liver, muscle, blood or hair samples of 31 specimens of *Cyclopes didactylus* from the Peruvian Departments of Ucayali (n = 2) and Loreto (n = 2), Suriname (n = 1), Colombia (n = 1) and the Brazilian States of Acre (n = 1), Amapá (n = 1), Amazonas (n = 5), Maranhão (n = 3), Pará (n = 6), Pernambuco (n = 4), Piauí (n = 3 - new occurrence record; F. R. Miranda, unpublished data), Rio Grande do Norte (n = 1) and Rondônia (n = 1) were collected since 2005 by the Institute of Research and Conservation of Anteaters in Brazil (Projeto Tamanduá) or obtained from other museums and institutions, and deposited at Laboratório de Biodiversidade e Evolução Molecular (LBEM) in Universidade Federal de Minas Gerais (UFMG), Brazil. The samples were preserved in 70% ethanol and the DNA extraction was performed, according to reagents availability, by a standard phenol-chloroform protocol (Sambrook and Russell, 2001) or using a DNeasy Blood & Tissue Kit (QIAGEN) following the manufacturer's instructions. A sequence from a French Guiana individual was retrieved from GenBank (accession number KT818539) for some analyses. A map of sampling localities and a detailed list of samples are available in Figure 1 and Table S1, respectively.

### Amplification and sequencing

Fragments of the mitochondrial control region (CR) and the Cytochrome c Oxidase subunit I (*COI*) and Cytochrome b (*Cyt-b*) genes were amplified with primers L_0_ [L15445] (Douzery and Randi, 1997) and E_3_ [H15978] (Huchon *et al.*, 2001), LCO1490 and HCO2198 (Folmer *et al.*, 1994), and CytB-L = 5'-CCATGAGGACAAATATCATTCTGAGG-3' and CytB-H = 5'-TGGTTTACAAGACCAGTGTAAT-3' (previously designed by our laboratory), respectively. Amplification reactions were carried out in a final volume of 10 μL containing 10 ng of DNA, 1 x reaction buffer (Invitrogen), 1.5 mM MgCl_2_ (Invitrogen), 100 μM dNTPs, 0.2 μM of each primer (forward and reverse), 0.5 mg/mL of BSA adjuvant and 0.2 U of Platinum® *Taq* DNA Polymerase (Invitrogen). Cycling reactions consisted of an initial denaturation step of 94 °C for 5 min, followed by 35 cycles of 94 °C for 30 s, 50 °C (*COI*), 52 °C (CR) or 53 °C (*Cyt-b*) for 45 s, 72 °C for 1 min, and a final extension step of 72 °C for 10 min. Adjustments in PCR reagents and template DNA concentrations and in primers annealing temperatures were made when necessary. PCR efficiency was assessed by electrophoresis on 1% agarose gel and the amplicons were submitted to purification protocol by polyethylene glycol 20% precipitation (described in Santos Júnior *et al.*, 2015). Purified amplicons were sequenced in a MegaBACE 1000 DNA Sequencing System (Amersham-Biosciences) or in an ABI 3130xl Genetic Analyzer (Applied Biosystems).

### Data analysis

Consensus sequences were generated with Phred v. 0.20425 (Ewing and Green, 1998; Ewing *et al.*, 1998), Phrap v. 0.990319 (Green, 1994-1999) and Consed 19.0 (Gordon *et al.*, 1998) or SeqScape v. 2.6 (Applied Biosystems) and aligned in MEGA 7 (Kumar *et al.*, 2016).

The concatenated sequences of the three mitochondrial fragments were used to construct a median-joining haplotype network (Bandelt *et al.*, 1999) using the NETWORK 5 software (Fluxus Technology Ltd, 1999-2016) to visualize the relationships between haplotypes and their geographical distribution. Also an analysis of molecular variance (AMOVA; Excoffier *et al.*, 1992) was performed in Arlequin v.3.5 (Excoffier and Lischer, 2010) to assess the distribution of genetic variability at different hierarchical levels. For the latter analysis, we included only the haplotype network clusters containing two or more samples and tested for significance with 10,000 permutations (*P* < 0.05).

To infer on the evolutionary history of the species, a molecular dating analysis was carried out using the BEAST 2.3 package (Bouckaert *et al.*, 2014). In this analysis, CR sequences were not considered, and both *COI* and *Cyt-b* sequences were partitioned by codon position. The analysis was performed using the reversible-jump based substitution model (Bouckaert *et al.*, 2013), allowing for gamma rate heterogeneity and invariant sites, and a relaxed clock log-normal model with a birth-death tree prior combined with soft fossil calibration constraints (Yang and Rannala, 2006). Calibration intervals for crown xenarthran nodes were based on Meredith *et al.* (2011) and are available in Table S2. Sequences of *Myrmecophaga tridactyla* (KT818549), *Tamandua mexicana* (KT818551), *Tamandua tetradactyla* (KT818552), *Bradypus torquatus* (KT818524), *Choloepus didactylus* (KT818537) and *Dasypus kappleri* (KT818541) retrieved from GenBank were used as outgroups. Three independent MCMC chains were run for 50,000,000 generations and sampled every 5,000 generations. Trace files were checked for chain convergence and sufficient effective sample sizes (ESS) in Tracer v. 1.6 (Rambaut *et al.*, 2014) and the tree files were combined in LogCombiner with a 50% burn-in. The maximum clade credibility (MCC) tree and the associated posterior probabilities and common ancestor heights were summarized with a 33% burn-in in TreeAnnotator from the 15,000 combined trees sampled from the three independent runs. BEAST 2.3 runs were carried out on CIPRES Science Gateway v.3.3 (Miller *et al.*, 2010).

## Results

### Sequencing

CR sequences varied in length from 299 to 308 bp due to indels (309 bp alignment; KU596973-KU597000), *COI* sequences of 555 bp (KU597001-KU597027) and *Cyt-b* sequences of 678 bp (KU597028-KU597057) were obtained for 28, 27 and 30 individuals, respectively. All of the specimens had at least one of the fragments sequenced, but only the 25 individuals that presented sequences for the three fragments were considered in the network and AMOVA with no gaps allowed. However, individuals with missing *COI* or *Cyt-b* sequences were included in the molecular dating analysis.

### Genetic structure

**Figure 2:**
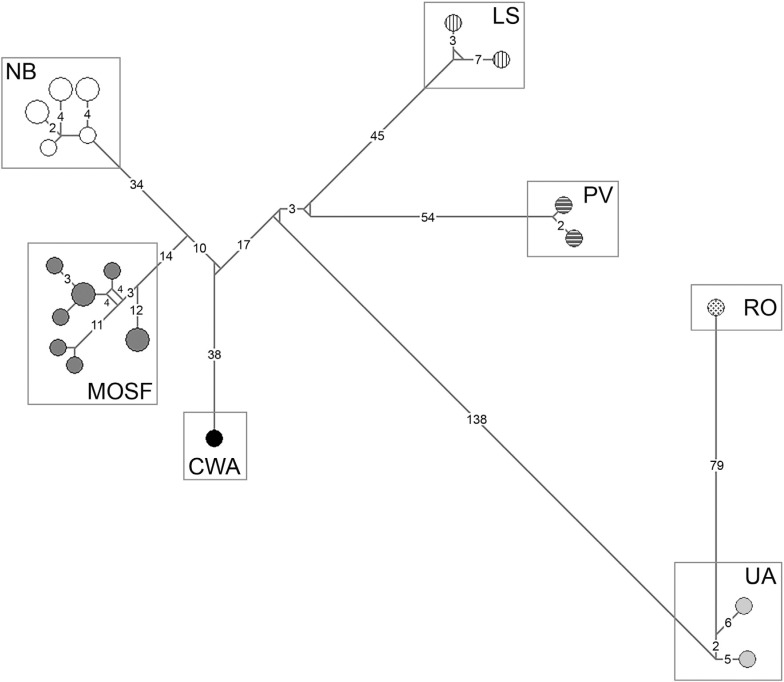
Mitochondrial haplotype network showing seven geographic clusters. The network was constructed with concatenated mitochondrial data using the median-joining algorithm. Circle sizes are proportional to frequencies, and mutation step numbers greater than one are indicated on the lines. UA = Ucayali; RO = Rondônia; LS = Loreto and Santa Isabel do Rio Negro; PV = Porto de Moz and Vitória do Xingu; CWA = Colombia to the west of the Andes; MOSF = Manaus, Oriximiná, Suriname and French Guiana; NB = northeastern Brazil.

The haplotype network revealed 20 mitochondrial haplotypes grouped in seven geographic clusters separated by a large number of mutations (Figure 2). These clusters correspond to haplotypes found in: Ucayali and Acre (UA cluster), Rondônia (RO cluster) and Porto de Moz and Vitória do Xingu (PV cluster), all located on the right bank of the Amazon River; Loreto and Santa Isabel do Rio Negro (LS cluster) and Manaus, Oriximiná, Suriname and French Guiana (MOSF cluster), all located on the left bank of the Amazon River; Colombia to the west of the Andes (CWA cluster); and Maranhão, Pernambuco and Rio Grande do Norte in northeastern Brazil (NB cluster). There were neither predominant nor shared haplotypes between clusters showing a marked genetic structure according to spatial distribution. The largest amount of mutation steps (> 138) occurred between two groups of haplotype clusters that coincided with a south-north division of the species distribution: the UA and RO clusters to the south and all the others to the north. Besides, the RO and CWA clusters comprised only one individual each and, thus, were excluded from the following AMOVA.

The AMOVA results for the five clusters analyzed (UA, LS, PV, MOSF and NB) reinforced the genetic structure exhibited in the haplotype network with a φ_ST_ estimate of 0.904 (*P* = 0.00000) indicating that most part of the genetic diversity of *C. didactylus* is due to differences between the clusters.

### Molecular dating

**Figure 3:**
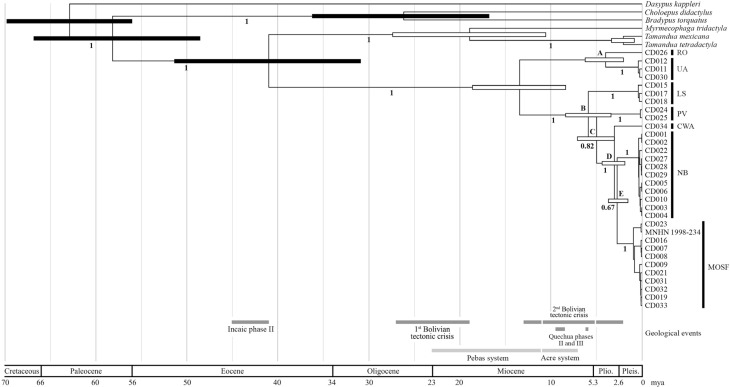
Molecular timescale for *Cyclopes didactylus* and other xenarthran nodes inferred in this study. Node ages were obtained using a relaxed clock model under the reversible-jump based substitution model, allowing gamma rate heterogeneity and invariant sites, and a birth-death tree prior with soft fossil calibrations. Node bars indicate the 95% HPD intervals for age estimates in million years ago. Plain black and white node bars indicate constrained and unconstrained nodes, respectively. Divergence dates less than 1 mya are not represented. Letters at nodes refer to Table 1.

The MCC tree obtained in the molecular dating analysis (Figure 3) showed a topology congruent with previous molecular phylogenetic studies involving xenarthran genera (Delsuc *et al.*, 2001, 2002, 2003, 2012; Möller-Krull *et al.*, 2007). In addition, it revealed two major monophyletic clades comprising seven mitochondrial lineages within *Cyclopes didactylus*, all fully supported, that corresponded to the south-north division of the species range and the seven geographic clusters found on the haplotype network, respectively. The phylogenetic relations between these mtDNA lineages were also supported by high posterior values (≥ 0.82) in all branches except the one grouping MOSF and NB as sister clusters (0.67). The individuals from Loreto (CD017), Acre (CD030), Amapá (CD016) and Manaus (CD032), and Piauí (CD027, CD028 and CD029), which were not included in the haplotype network, grouped in the phylogeny within the LS, UA, MOSF and NB lineages, respectively, according to our expectations. Furthermore, our time estimate (Figure 3 and Table 1) is compatible with previous molecular dating studies (Delsuc *et al.*, 2004, 2012; Gibb *et al.*, 2016). The only notable difference regards Myrmecophagidae and *Tamandua* nodes. For these nodes, the newly estimated ages, 19 and 2 mya, respectively, are considerably older than previous ones, 13-10 and 1 mya, respectively (Delsuc *et al.*, 2004, 2012; Gibb *et al.*, 2016). Such differences are expected with a denser taxon sampling as reported by Gibb *et al.* (2016) for Folivora, Dasypodinae, Euphractinae and Tolypeutinae nodes.

**Table 1:** Divergence time estimates for *Cyclopes didactylus* and other xenarthran nodes inferred in this study. Node ages were obtained using the reversible-jump based substitution model with gamma rate heterogeneity and invariant sites and a relaxed clock model. Mean posterior estimates and 95% HPD intervals are expressed in million years ago. Divergence dates less than 1 mya are not shown. Letters A - E refer to nodes in Figure 3.

Node	Mean	Min.	Max.
Xenarthra a	62.89	55.68	69.91
Pilosa a	58.15	48.46	66.96
Folivora a	26.18	16.81	36.13
Vermilingua a	40.99	30.9	51.3
Myrmecophagidae	18.92	10.64	27.51
*T. mexicana* / *T. tetradactyla*	2.06	0.87	3.46
*Cyclopes didactylus*	13.45	8.34	18.82
A	3.97	1.94	6.27
B	5.88	3.43	8.42
C	4.97	3.0	7.08
D	3.05	1.78	4.48
E	2.72	1.39	3.78

a fossil calibrated nodes

## Discussion

### Phylogeographic patterns in *Cyclopes didactylus*

The mtDNA sequences of the silky anteater analyzed here revealed the existence of two lineages (UA and RO) located in the southern part of the species' current range and five lineages (LS, PV, CWA, MOSF and NB) present in its northern part. This pattern of south-north division is supported by the largest amount of mutation steps (> 138) found between those haplotype clusters in the network (Figure 2) and by the oldest divergence (13.45 mya) within *C. didactylus* in the dated phylogeny (Figure 3 and Table 1). Even though we could not determine a clear geographic boundary for this major genetic division, it indicates an initial diversification close to the Andes. In addition, the UA, RO and PV lineages seem to be geographically isolated from the LS and MOSF lineages by the Solimões/Amazon Rivers. Moreover, there is a phylogeographic break between LS and MOSF lineages, which may be represented by the Negro River acting as a barrier. Although the Solimões/Amazon and Negro Rivers apparently limit the range of some of the mtDNA lineages of *C. didactylus*, they may not be historical boundaries or causal mechanisms for the divergences.

The CWA lineage is isolated from the Amazonian silky anteaters by the eastern Andean Cordillera in northern Colombia. This lineage was probably already there when the eastern Andes was no more than 40% of its modern elevation (Gregory-Wodzicki, 2000). Thus, the mountain uplift in that region between 5 and 2 mya (Lundberg *et al.*, 1998; Gregory-Wodzicki, 2000; Hoorn *et al.*, 2010) may be likely the vicariant event responsible for its isolation and differentiation at ca. 3.05 mya. This pattern of west and east divergence in the Andean region is also observed for the sloths *B. variegatus* and *C. hoffmanni* (Moraes-Barros and Arteaga, 2015), woodcreepers of the genus *Dendrocincla* (Weir and Price, 2011) howler monkeys (*Alouatta*; Cortés-Ortiz *et al.*, 2003), and bats (Redondo *et al.*, 2008).

The haplotypes from the Brazilian Atlantic forest formed a monophyletic group (NB) with the haplotypes from Maranhão and Piauí suggesting a past connection between the Amazon and the Atlantic forests, at least 2.72 mya, when this lineage diverged. Such connection through northeastern Brazil was also suggested for phylogeographic patterns of woodcreeper species endemic to the Atlantic forest (Cabanne *et al.*, 2008; Weir and Price, 2011) and small mammals (Costa, 2003).

From an evolutionary standpoint, the first lineages of silky anteater to diverge (UA-RO and LS) are found in Western Amazonia, and those diverging more recently are found in Eastern Amazonia (PV and MOSF) and the Atlantic forest (NB), or to the west of the Andes in northwestern South America (CWA). This suggests that *C. didactylus* mitochondrial lineages originated in Western Amazonia, somewhere around Peru.

The large amount of mutation steps separating groups of exclusive haplotypes, which corresponded to monophyletic lineages, and the high degree of genetic differentiation (φ_ST_ = 0.904) between at least five of these groups (UA, LS, PV, MOSF and NB), not only shows that *C. didactylus* is genetically and geographically structured, but also that the populations and their mtDNA lineages are very distinct from each other. However, such remarkable genetic structure disagrees with the proposed subspecies division (Wetzel, 1982; Gardner, 2007), where five of them should be represented in our sampling based on their ranges (Hayssen *et al.*, 2012): *C. d. catellus* (Rondônia/Brazil), *C. d. didactylus* (Pernambuco-Rio Grande do Norte/Brazil, Suriname and French Guiana), *C. d. dorsalis* (Colombia), *C. d. ida* (Ucayali-Loreto/Peru and Acre-Amazonas/Brazil) and *C. d. melini* (Amazonas-Pará-Amapá-Maranhão/Brazil). This raises the need to review the current taxonomic status of *Cyclopes didactylus* (F. R. Miranda *et al.*, in prep.).

### Molecular dating of xenarthran lineages

Our age estimates corroborate the general findings about xenarthrans, which first appeared in the early Paleocene and subsequently underwent an impressive radiation during the Tertiary, when South America was isolated from other landmasses (Delsuc *et al.*, 2004). Major tectonic and climatic events were already associated with the diversification of sloths, anteaters and armadillos (Delsuc *et al.*, 2004; Moraes-Barros and Arteaga, 2015; Gibb *et al.*, 2016). However, a few discrepancies regarding previous time estimates (Delsuc *et al.*, 2004, 2012; Gibb *et al.*, 2016) were observed for divergences within Myrmecophagidae, and can be explained by the increased number of *Cyclopes didactylus* samples used in our analysis. As previously said, this is expected with a denser taxon sampling (Gibb *et al.*, 2016), which affects the coalescence between myrmecophagid species causing them to become older: around 19 mya compared to 13-10 mya (Delsuc *et al.*, 2004, 2012; Gibb *et al.*, 2016) for the divergence between *Myrmecophaga* and *Tamandua*, and around 2 mya compared to 1 mya (Gibb *et al.*, 2016) for the separation between *T. tetradactyla* and *T. mexicana*. The myrmecophagid splits are discussed below.

The divergence between *Myrmecophaga tridactyla* and *Tamandua* at ca. 19 mya correlates well with the end of the first Bolivian tectonic crisis in the early Miocene (27-19 mya; Sempere *et al.*, 1990). This period also coincides with a global warm phase, which culminated in the Middle Miocene Climatic Optimum (Zachos *et al.*, 2001). Such events were already associated with the diversification of modern sloths lineages and Tolypeutinae armadillos (Delsuc *et al.*, 2004).

The age for separation between *T. mexicana* and *T. tetradactyla* at ca. 2 mya matches up with the ending of the final uplift of the Northern Andes (5-2 mya; Lundberg *et al.*, 1998; Hoorn *et al.*, 2010). Thus, this geologic event may explain the vicariance seen between the two *Tamandua* species, as suggested by Gibb *et al.* (2016).

### Insights on the evolutionary history of *Cyclopes didactylus*

The split of lineages separating *Cyclopes* and the other anteaters genera occurred around 41 mya, in the middle Eocene, shortly after a large episode of mountain uplift in the Andes of Peru known as “Incaic tectonic phase II”, estimated between 45 and 41 mya (Noble *et al.*, 1990; Lundberg *et al.*, 1998; Delsuc *et al.*, 2004; Antonelli *et al.*, 2009). After that, an interval of more than 27 million years preceded the start of the silky anteater diversification.

The first divergence within *Cyclopes didactylus*, at ca. 13.5 mya, separated two major monophyletic clades: one that would give rise to the UA and RO lineages, in the south of the species current distribution; and other that would originate all the other lineages, in the north. This connects with the start of an intensified uplift in the Central and Northern Andes between 13 and 11 mya (Antonelli *et al.*, 2009; Hoorn *et al.*, 2010) and with the final stages of the so-called “Pebas” system (Hoorn *et al.*, 2010). From 23 to 11 mya, this system transformed most of Western Amazonia in a large wetland of shallow lakes and swamps that fragmented the preexisting rainforest (Antonelli *et al.*, 2009; Hoorn *et al.*, 2010). For an arboreal species with low dispersal abilities like the silky anteater, a fragmented forest habitat could promote isolation for a period of time sufficient for the divergence between the southern and northern clades.

From 11 to 7 mya, parallel to a new period of rapid Andean mountain building sometimes termed “Quechua phases II and III” (Mégard, 1984; Noble *et al.*, 1990; Antonelli *et al.*, 2009), the lacustrine Pebas system changed into a fluvial or fluviotidal “Acre” system (Hoorn *et al.*, 2010). The return of forested habitats shortly after the demise of Western Amazonian wetlands (Hoorn *et al.*, 2010) may have triggered the divergence of the mitochondrial lineage found in Loreto and Santa Isabel do Rio Negro (LS) at ca. 6 mya. Similarly, plant diversity also increased between 7 and 5 mya, following the return of terrestrial conditions (Hoorn *et al.*, 2010).

The subsequent divergence between PV and the other mitochondrial lineages at around 5 mya, cannot be explained by vicariance and, thus, may have been prompted by other factors such as biotic interactions.

The haplotypes found in Ucayali and Acre (UA) and the one found in Rondônia (RO) became separated mitochondrial lineages around 4 mya. This period followed the end of the second major Bolivian tectonic crisis in the late Miocene (11-5 mya; Marshall and Sempere, 1991).

In the case of the Colombian haplotype (CWA), which is separated from the other South American lineages by the Northern Andes, the estimated divergence at ca. 3 mya correlates well, as previously said, with the end of the final uplift of the Eastern Cordillera in that region (Lundberg *et al.*, 1998; Gregory-Wodzicki, 2000; Hoorn *et al.*, 2010), as observed for the vicariance between the two *Tamandua* species.

Finally, the mitochondrial lineages found in northeastern Amazonia (MOSF) and northeastern Brazil (NB) diverged at 2.7 mya. The fact that the individuals from Maranhão coastline forest and Piauí mangrove, both areas within the main distribution of the species in South America, group together with the small population of the Atlantic forest suggests that these distribution areas were actually connected. In addition, the order of the divergences within the NB lineage is congruent with a dispersal event starting from Maranhão and crossing all the way to the northeastern Atlantic forest. A potential cause for this dispersal of *C. didactylus* could be associated with the beginning of the Pleistocene glacial cycles at around 2.6 mya (Hoorn *et al.*, 2010; Antonelli and Sanmartín, 2011) and a likely connection between Amazon and Atlantic forests (Costa, 2003; Auler *et al.*, 2004; Wang *et al.*, 2004; Cabanne *et al.*, 2008; Batalha-Filho *et al.*, 2013).

The small number of samples from CWA and PV lineages included in our molecular dating analysis, and sampling gaps like the one we have in southern Amazonia, between Purus and Tapajós rivers, hinders detailed interpretations on how dispersal events occurred and increases the possibility of unidentified lineages that would likely change the topology of the phylogeny and, consequentially, alter the sequence and/or age of the splits. We recognize the difficulty in obtaining samples of this elusive species, but future studies will need a denser and more extensive sampling to overcome these problems.

In summary, our results show a strong and complex genetic structure for the silky anteater population distribution, and confirm the antiquity of its lineage, which separated from the other vermilinguas as early as the middle Eocene (41 mya) and began to diversify in the late-middle Miocene (13.5 mya) in southwestern Amazonia. Moreover, we emphasize the importance of the tectonic and climatic changes that took place in South America during the Tertiary and Quaternary for the species diversification and population dynamics.

## Supplementary material

The following online material is available for this article:

Table S1 - Detailed list of samples and respective localities.

Table S2 - List of fossils used as soft calibration constraints in the molecular dating analysis.
