# Supplementary material for: Phylogeographic history of South American populations of the silky anteater Cyclopes didactylus (Pilosa: Cyclopedidae)
Source: Genet Mol Biol. 2017 Feb 13;40(1):40–9. doi: 10.1590/1678-4685-GMB-2016-0040 (PMC5409769; doi:10.1590/1678-4685-GMB-2016-0040)
Supplement: Supplementary file 1 [file 1415-4757-gmb-1678-4685-GMB-2016-0040-Suppl01.pdf]

Table S1 - Detailed list of samples and respective localities.

LBEM ID	Sample	Country	State/Department	Municipality	Latitude	Longitude	mtDNA sequences
M0978	CD001	Brazil	Maranhão	São Luís	02°35'56.33"S	44°13'51.32"W	CR, *COI*, *CytB*
M0979	CD002	Brazil	Maranhão	São Luís	02°35'56.33"S	44°13'51.32"W	CR, *COI*, *CytB*
	CD003	Brazil	Pernambuco	Igarassu	07°49'15.46"S	34°54'18.48"W	CR, *COI*, *CytB*
	CD004	Brazil	Pernambuco	Igarassu	07°45'12.82"S	34°59'49.48"W	CR, *COI*, *CytB*
	CD005	Brazil	Pernambuco	Timbaúba	07°34'35.37"S	35°21'46.47"W	CR, *COI*, *CytB*
	CD006	Brazil	Pernambuco	Jaboatão dos Guararapes	08°10'09.05"S	35°01'43.86"W	CR, *COI*, *CytB*
	CD007	Brazil	Pará	Oriximiná	01°25'34.06"S	56°25'36.82"W	CR, *COI*, *CytB*
	CD008	Brazil	Pará	Oriximiná	00°51'15.29"S	57°01'32.55"W	CR, *COI*, *CytB*
	CD009	Brazil	Pará	Oriximiná	01°32'09.67"S	56°07'42.33"W	CR, *COI*, *CytB*
	CD010	Brazil	Rio Grande do Norte	Goianinha	06°16'00.49"S	35°12'30.70"W	CR, *COI*, *CytB*
	CD011	Peru	Ucayali	Atalaya	09°51'07.72"S	73°02'44.86"W	CR, *COI*, *CytB*

	CD012	Peru	Ucayali	Purús	10°22'39.74"S	71°37'51.86"W	CR, *COI*, *CytB*
	CD015	Brazil	Amazonas	Santa Isabel do Rio Negro	00°30'42.24"S	64°59'44.02"W	CR, *COI*, *CytB*
	CD016 [a]	Brazil	Amapá	Macapá			*CytB*
	CD017 [a]	Peru	Loreto				CR, *CytB*
	CD018	Peru	Loreto	Maynas	03°53'55.53"S	73°17'39.70"W	CR, *COI*, *CytB*
	CD019	Brazil	Amazonas	Manaus	03°07'08.12"S	60°01'17.47"W	CR, *COI*, *CytB*
M0676	CD021	Brazil	Pará	Oriximiná	01°08'33.65"S	57°29'37.89"W	CR, *COI*, *CytB*
M1653	CD022	Brazil	Maranhão	Rosário	02°53'24.32"S	44°15'26.89"W	CR, *COI*, *CytB*
M1712	CD023	Suriname	Wanica	Houttuin	05°43'56.68"N	55°10'54.87"W	CR, *COI*, *CytB*
M1729	CD024	Brazil	Pará	Vitória do Xingu	03°01'24.97"S	52°01'56.28"W	CR, *COI*, *CytB*
M1730	CD025	Brazil	Pará	Porto de Moz	02°31'27.45"S	52°16'24.79"W	CR, *COI*, *CytB*
	CD026	Brazil	Rondônia	Espigão do Oeste	11°35'45.50"S	60°48'36.56"W	CR, *COI*, *CytB*
M2091	CD027 [a]	Brazil	Piauí	Ilha Grande			CR, *CytB*

M2090	CD028 [a]	Brazil	Piauí	Ilha Grande			*COI*, *CytB*
M2089	CD029 [a]	Brazil	Piauí	Ilha Grande			*COI*, *CytB*
	CD030 [a]	Brazil	Acre	Porto Walter			CR, *COI*
	CD031 [a]	Brazil	Amazonas	Manaus			CR, *COI*, *CytB*
	CD032 [a]	Brazil	Amazonas	Manaus			CR, *CytB*
	CD033 [a]	Brazil	Amazonas	Manaus			CR, *COI*, *CytB*
	CD034	Colombia	Santander	Girón	07°05'32.00"N	73°22'00.00"W	CR, *COI*, *CytB*
	MNHN 1998-234 [a b]	French Guiana					CR, *COI*, *CytB*

[a] exact coordinates unknown

[b] retrieved from GenBank (KT818539)
