# Supplementary material for: Phylogeographic history of South American populations of the silky anteater Cyclopes didactylus (Pilosa: Cyclopedidae)
Source: Genet Mol Biol. 2017 Feb 13;40(1):40–9. doi: 10.1590/1678-4685-GMB-2016-0040 (PMC5409769; doi:10.1590/1678-4685-GMB-2016-0040)
Supplement: Supplementary file 2 [file 1415-4757-gmb-1678-4685-GMB-2016-0040-Suppl02.pdf]

Table S2 - List of fossils used as soft calibration constraints in the molecular dating analysis. Minimum and maximum ages are according to Meredith *et al.* (2011) and are expressed in million years ago.

**Node**	**Fossil**	**Age**	
		**Min.**	**Max.**
Xenarthra	*Riostegotherium*	58.5	71.2
Pilosa	*Pseudoglyptodon chilensis*	31.5	65.5
Vermilingua	*Protamandua*	15.97	61.1
Folivora	*Megalonychidae*	15.97	40.6
